# A Rare Cause of Drug-Induced Pancytopenia: Trimethoprim–Sulfamethoxazole-Induced Pancytopenia

**DOI:** 10.3390/clinpract11020050

**Published:** 2021-06-03

**Authors:** Khalid Sawalha, Philip T. Sobash, Gilbert-Roy Kamoga

**Affiliations:** Internal Medicine Division, White River Health System, Batesville, AR 72501, USA; PSobash@wrmc.com (P.T.S.); RKamoga@wrmc.com (G.-R.K.)

**Keywords:** trimethoprim–sulfamethoxazole, pancytopenia, drug toxicity

## Abstract

Pancytopenia is a decrease across cellular hematological lines. Many different etiologies can cause this clinical picture including viral and bacterial infections, chemicals, malignancy, and medications. Particular attention should be paid to the onset, timing, and severity as they can indicate the underlying cause. In cases of iatrogenic-induced pancytopenia, the offending agent should be stopped immediately and the patient should be monitored for recovery of cell lines. While not well reported in the literature, trimethoprim–sulfamethoxazole (TMP-SMX) is a cause of pancytopenia. We present a case of drug-induced pancytopenia secondary to TMP-SMX that resolved quickly with cessation of use.

## 1. Introduction

Pancytopenia refers to decreases in all peripheral blood lineages and is considered to be present when all three cell lines are below the normal reference ranges as published by the World Health Organization: red blood cells with hemoglobin <12 g/dL for nonpregnant women and <13 g/dL for men, white blood cells <11 K/uL, and platelets <150,000 K/uL. Threshold levels for these lines may differ in value may differ depending on age, sex, and race [1]. Pancytopenia can be associated with a multitude of disease states including bacterial or viral infections, liver disease, malignancy, and iatrogenic causes. A thorough history and physical examination along with focused laboratory studies are necessary to establish a diagnosis and select proper management. We present a case of trimethoprim–sulfamethoxazole (TMP-SMX)-induced pancytopenia.

## 2. Case Presentation

We present a 62-year-old female with past medical history of bipolar depression and diabetes mellitus type II well controlled. She initially presented to our emergency department with nonspecific symptoms of overall lethargy, weakness, and fatigue. She was recently hospitalized during a manic episode. At this time, she was started on lithium, in addition to her home Seroquel. Of note, her CBC on discharge was within normal limits, 12 days before current admission. TMP-SMS and lithium were held on admission.

During the time between admission for manic episode and present, the patient stated she believed she had a urinary tract infection with some pain on urination and leakage of urine, for which she received TMP-SMX. She was unsure of the dosage but stated she had taken it for 5 days and finished 3 days before admission. On admission, she was noted to be febrile at 101.2, with a WBC of 3.1 K/μL, Hgb 9.4 g/dL, and Plt 84 K/μL. She did not complain of shortness of breath but had vague abdominal pain and pallor on exam. Chest X-ray showed no focal consolidations. Peripheral smear, LDH, and uric acid were ordered upon admission and can be seen in Table 1, along with further laboratory workup. With her symptoms appearing to be viral in nature, antibiotics were not started on admission, but tests for influenzae and COVID-19 were ordered which returned negative.

On D1 of admission, the patient had a Tmax of 102.7 and became hypotensive. She was transferred to the ICU. A central line was placed and broad-spectrum antibiotics, vancomycin protocol, meropenem, and doxycycline, were initiated. Norepinephrine infusion was started after her hypotension did not respond to intravenous fluid bolus. The norepinephrine was weaned off within 36 h due to her worsening status after admission; tick and viral panels were sent as in Table 1. With concern of unknown infectious source, a CT abdomen was performed, and it was unremarkable.

With workup being negative thus far and some improvement noted, it was decided to continue on antibiotics and monitoring of her blood cell counts. The prevailing thought at this point in time was lithium versus TMP-SMX use, which had been held on admission, since her counts had decreased objectively at some point after initiation of these drugs. Her hematopoietic cell lines took an upward trend on D3 of admission, with continuing uptrend thereafter. Due to her increasing counts, the antibiotics were stopped one at a time with vancomycin on day 4 and then meropenem on day 5. She completed a 10-day course of doxycycline for possible tick-borne illness since the tick panels had not yet resulted by day 10. Her blood cultures remained negative; respiratory viral panel and tick panel along with other laboratory evaluation identified no bacterial or viral etiology.

## 3. Discussion

Hematopoiesis (blood cell production) in the healthy adult takes place in the bone marrow, from which mature blood cells migrate into the circulation, spleen, and other sites. The bone marrow is dynamic and serves as a hematopoietic reservoir that responds to ongoing needs for cell production. The levels of circulating blood cells are determined by a balance between production; distribution in other organs; and ongoing cellular destruction such as white blood cells fighting infections, platelet consumption in blood clots, and cellular senescence [2,3,4,5]. 

Broadly speaking, pancytopenia may be caused by one or more of the following mechanisms: bone marrow infiltration (such disorders may include hematologic malignancies including leukemia, lymphoma, multiple myeloma, myelodysplastic syndromes, metastatic cancer, myelofibrosis, and infectious diseases such as miliary tuberculosis and fungal infections) [1,2]; bone marrow aplasia with nutritional disorders including deficiencies of vitamin B12 or folate, aplastic anemia, infectious diseases (e.g., HIV, viral hepatitis, parvovirus B19, immune destruction, and medications are among the causes of marrow aplasia) [3]; and blood cell destruction, as occurs in disseminated intravascular coagulation, thrombotic thrombocytopenic purpura, and ineffective hematopoiesis, while excessive sequestration may be due to hypersplenism (e.g., from liver cirrhosis, storage diseases, lymphoma, or autoimmune disorders).

When a particular medication is suspected as a cause of pancytopenia, it should be discontinued promptly. This decision will be influenced by the severity of the pancytopenia, trajectory of the blood counts, clinical symptoms, and the necessity of medication.

TMP-SMX, also known as co-trimoxazole, is a bacteriostatic antibiotic with a combination of two antimicrobial agents. These agents act synergistically against a wide variety of bacteria. TMP is a structural analog of para-aminobenzoic acid (PABA), and this structural similarity results in competitive inhibition with PABA to inhibit the synthesis of dihydrofolic acid, an intermediate step in the formation of tetrahydrofolate (THF) [6,7]. TMP binds to bacterial dihydrofolate reductase, also preventing the formation of THF [8]. While known to inhibit dihydrofolate reductase (DHFR) in bacteria, it does not affect mammalian cells. Most cases of hematological cellular lines being affected with TMP-SMX are with thrombocytopenia [9]. Pancytopenia is a less well demonstrated side effect, and the mechanism is currently unknown. While some studies have demonstrated an acute reduction in folate when DHFR inhibitors are used, this does not explain the acuity of pancytopenia seen [10]. Our case also demonstrated no decrease in folate. There may be some element of DNA disruption causing acute lysis, but this is unclear. 

**Figure 1:**
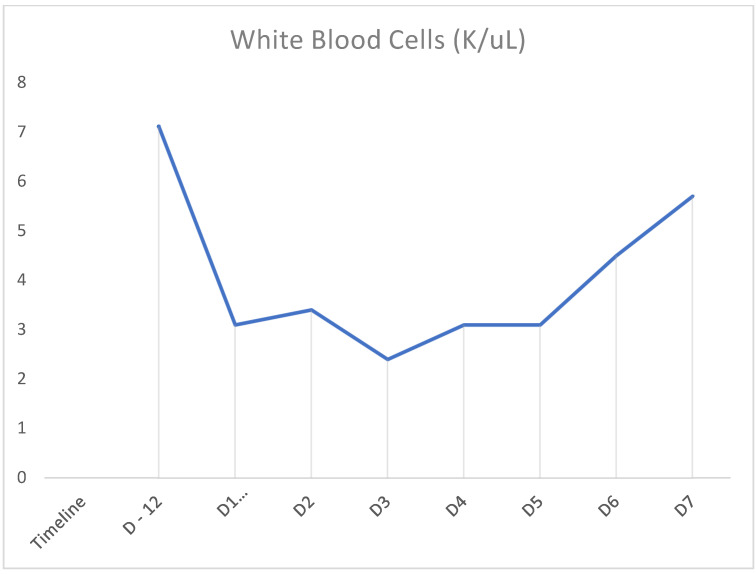
Timeline demonstrating white blood cell counts on prior admission (D-12) and rise in counts after cessation of TMP-SMX on D1 of admission.

**Figure 2:**
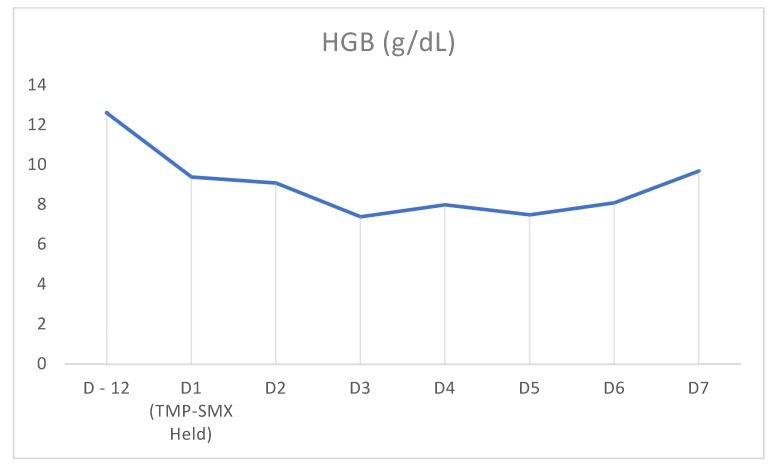
Timeline demonstrating hemoglobin counts on prior admission (D-12) and rise in counts after cessation of TMP-SMX on D1 of admission.

**Figure 3:**
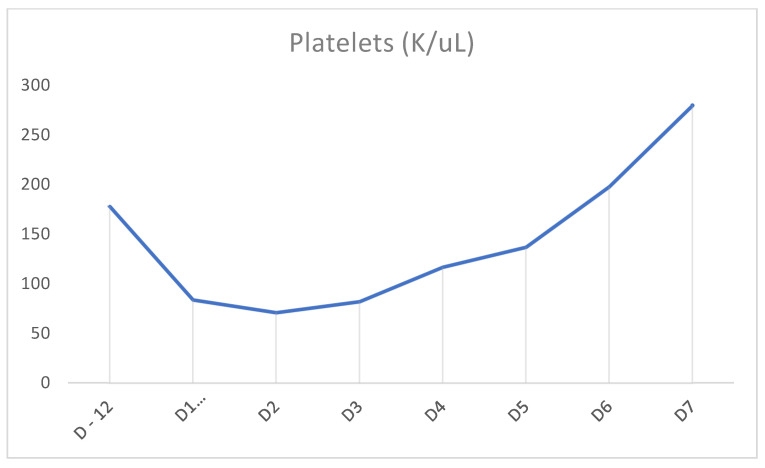
Timeline demonstrating platelet cell counts on prior admission (D-12) and rise in counts after cessation of TMP-SMX on D1 of admission.

In our case above, the patient had received TMP-SMX before presenting with pancytopenia. Although history and physical examination did not reveal an underlying cause, there was a strong suspicion that recent TMP-SMX use was the cause. Other etiologies were ruled out through peripheral blood smear; tick panel; folic acid and vitamin B12 levels; and viral panel including hepatitis, CMV, EBV, and HIV. Bone marrow biopsy was not performed as the patient’s counts improved. Once TMP-SMX was discontinued, her blood counts progressively improved within the following days, as can be seen in Figure 1, Figure 2 and Figure 3. It should also be noted that the patient was not started on B12 prior to discharge from the hospital. We also believe that even if this deficiency had been addressed at this time, it would not have taken effect in the time frame of our case. Furthermore, this deficiency is known to cause thrombocytopenia and anemia more often than pancytopenia.

## 4. Conclusions

History and physical examination should consider the severity and time course of pancytopenia and associated symptoms. When extensive workup yields no identifiable cause, iatrogenic causes should be further explored. While TMP-SMX is known to cause pancytopenia, it is not widely reported in the literature, and further evaluation should be sought to reveal more about the mechanism behind it. 

## Figures and Tables

**Table 1:** Laboratory workup with results for presentation of pancytopenia.

Pancytopenia Initial Laboratory Workup	Values	Reference Range
Reticulocyte Count	3.15%	0.6–2.3%
Neutrophil %	85%	40–70%
White Blood Cell	3.1 K/μL	4.5–11
Hemoglobin	9.4 g/dL	13.5–18
Platelet	84 K/μL	150–450
Fibrinogen	438 md/dL	207–442 mg/dL
D-dimer	2.22 ug/mlFEU	0–0.48 ug/mlFEU
Lactic Acid	0.9 mmol/L	0.7–2 mmol/L
Uric Acid	4.8 mg/dL	2.5–6.2 mg/dL
Lactate Dehydrogenase	576 U/L	313–618 U/L
Vitamin B12	169 pg/mL	230–931 pg/mL
Folate	7.7 ng/mL	2.70–19 ng/mL
Procalcitonin	1.22 ng/mL	<0.5 ng/mL
Lithium	0.6 mmol/L	0.6–1.2 mmol/L
Tick Panel	Negative	
Hepatitis Panel	Negative	
COVID-19	Negative	
Influenzae	Negative	
Blood Cultures	Negative	
CMV	Negative	
EBV	Negative	

## Data Availability

Data available upon request.
